# More trust in networks, more secure keys

**DOI:** 10.1038/s41377-024-01693-x

**Published:** 2025-01-02

**Authors:** Guan-Jie Fan-Yuan, Shuang Wang

**Affiliations:** 1 CAS Key Laboratory of Quantum Information, University of Science and Technology of China, 230026 Hefei, China; 2 Anhui Province Key Laboratory of Quantum Network, University of Science and Technology of China, 230026 Hefei, China; 3 CAS Center for Excellence in Quantum Information and Quantum Physics, University of Science and Technology of China, 230026 Hefei, China; 4 Hefei National Laboratory, University of Science and Technology of China, 230088 Hefei, China

**Keywords:** Fibre optics and optical communications, Quantum optics

## Abstract

A novel continuous-variable quantum passive optical network is proposed in which a user can increase their key rate by trusting other users. This is because the keys, which would be discarded to remove correlations with untrusted users, can be retained when the users are trusted. It provides a new perspective for enhancing network performance.

Quantum key distribution (QKD)^1^, as a quantum communication technology that provides point-to-point information-theoretic secure key sharing, must consider how to extend its native point-to-point system into a network architecture that supports fully connected, all-time communication for large-scale applications. Networking requires addressing three key issues in sequence: connectivity, stability, and high throughput. To establish connections, one approach is an active optical network using, for example, optical switches^2–4^, which connect different pairs of users based on control signals. The other approach is a passive optical network (PON), which connects a group of users through components like splitters^5,6^ and demultiplexers^7,8^. Heterogeneous integration is more flexible and mature^9^. Some methods, for example, self-stabilizing encoding^10^, active compensation^11^ and passive matching^12^, can achieve high stability in the presence of environmental disturbance.

On this basis, increasing the key rate is necessary to support more services. The most direct approach is to increase the repetition rate, enhance the fidelity of quantum state encoding and decoding, and improve detector performance. These methods essentially enhance the fundamental performance of point-to-point systems, but things become more complex within a network. The passive network with one transmitter and multiple receivers, an architecture suitable for access networks, allows quantum states from the same sender to be measured by multiple receivers, which may lead to correlations between the keys of the users. For security, each user must compress their keys, thereby removing this correlation. For discrete-variable QKD, this can be naturally satisfied when extracting single-photon information, as a single photon can only collapse onto one user’s detector, thus avoiding any correlations. For continuous-variable QKD (CV-QKD), it is necessary to address this during the privacy amplification process.

Imagine a scenario where a user claims to have removed the correlation between their key and the other user’s key. If their claim is accepted, then the other user would no longer need to remove the portion of their key that is correlated with this user, as the correlation is mutual. In a recent paper published in *Light: Science & Applications*, Hajomer et al. from the Technical University of Denmark and Filip et al. from the Palacky University have proposed a CV-QKD PON to increase secure keys by trusting other users^13^. In this protocol, the transmitter (referred to as the provider) sequentially conducts key distillation with each receiver (referred to as the user). The first user chooses to distrust all other users in the network. The second user, however, chooses to trust the first user, and so on, allowing each subsequent user to trust users who have already completed distillation (see Fig. 1). They demonstrated the advantage of this protocol in an eight-user network experiment. Considering the finite-size effect, the network’s total key rate reaches 1 Mbps with an average distance of 11 km. In comparison, the key rate for an untrusted protocol is 0.4 Mbps.

**Fig. 1:**
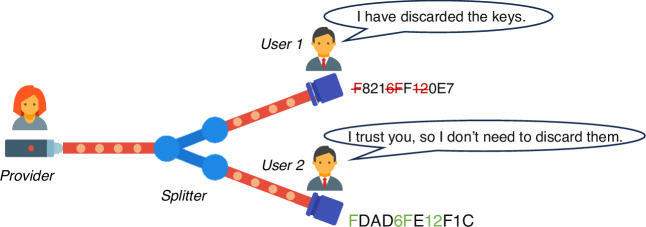
Schematic diagram of the untrusted protocol. The homogeneity of signals associates the users’ keys. Users in the network sequentially distil their keys. If a subsequent user trusts that the previous users have discarded the correlated keys, then they can retain those keys. The example of the keys is illustrative, not strict, because key compression is a mapping of bit strings rather than simply deleting certain bits

This work finds a new key-rate mine: trust. Based on reliable identity authentication, this scheme is very useful for scenarios of group interaction such as meetings. Moreover, it further clarified the relationship between security and performance, providing additional references for standardization and application. This encourages us to reassess the role of network trustworthiness in quantum networks, rather than merely striving for minimal assumptions, especially in the context of practicality. Following this, future research may find additional points for increasing key rates and clarify the trade-offs in different application scenarios.
